# Cognitive profile in cerebral small vessel disease: comparison between cerebral amyloid angiopathy and hypertension-related microangiopathy

**DOI:** 10.1038/s41598-024-55719-w

**Published:** 2024-03-11

**Authors:** Eleonora Barucci, Emilia Salvadori, Simona Magi, Martina Squitieri, Giulio Maria Fiore, Lorenzo Ramacciotti, Benedetta Formelli, Francesca Pescini, Anna Poggesi

**Affiliations:** 1 NEUROFARBA Department, Neuroscience Section, University of Florence, Careggi University Hospital, Largo Brambilla 3, 50134 Florence, Italy; 2 Department of Biomedical and Clinical Sciences, University of Milan, Milan, Italy; 3 Stroke Unit, Careggi University Hospital, Florence, Italy

**Keywords:** Cerebral small vessel disease, Cerebral amyloid angiopathy, Microangiopathy, Cognitive decline, Cognitive profile, Cognitive impairment, Neuropsychological patterns, Cognition, Arteriolosclerosis, Neuroscience, Psychology

## Abstract

Cerebral amyloid angiopathy (CAA) is recognized as a cause of cognitive impairment, but its cognitive profile needs to be characterized, also respect to hypertension-related microangiopathy (HA). We aimed at comparing difference or similarity of CAA and HA patients’ cognitive profiles, and their associated factors. Participants underwent an extensive clinical, neuropsychological, and neuroimaging protocol. HA patients (n = 39) were more frequently males, with history of vascular risk factors than CAA (n = 32). Compared to HA, CAA patients presented worse performance at MoCA (p = 0.001) and semantic fluency (p = 0.043), and a higher prevalence of amnestic MCI (46% vs. 68%). In univariate analyses, multi-domain MCI was associated with worse performance at MoCA, Rey Auditory Verbal Learning Test (RAVLT), and semantic fluency in CAA patients, and with worse performance at Symbol Digit Modalities Test (SDMT) and phonemic fluency in HA ones. In multivariate models, multi-domain deficit remained as the only factor associated with RAVLT (β = − 0.574) in CAA, while with SDMT (β = − 0.364) and phonemic fluency (β = − 0.351) in HA. Our results highlight different patterns of cognitive deficits in CAA or HA patients. While HA patients’ cognitive profile was confirmed as mainly attentional/executive, a complex cognitive profile, characterized also by deficit in semantic memory, seems the hallmark of CAA patients.

## Introduction

Cerebral Small Vessel Disease (cSVD) is a term used to describe a group of pathological processes having different etiology and pathogenesis that affect the small vessels of the brain, i.e. small arteries, arterioles, capillaries, and small veins^1^. The consequences of cSVD on the brain parenchyma are mainly subcortical lesions: white matter hyperintensities (WMH) of presumed vascular origin, lacunes, cerebral microbleeds (CMBs), enlarged perivascular spaces (EPVS) and microinfarcts^1–4^. These lesions might be clinically silent (covert cSVD), may manifest with an acute stroke, either ischemic or hemorrhagic, and/or determine progressive cognitive, functional and motor worsening due to increasing lesion burden^1^. In fact, cSVD is widely recognized as a leading cause of stroke, disability, cognitive impairment, dementia, and functional loss in the elderly.

Rather than a homogeneous disorder, cSVD is a spectrum of diseases encompassing different sporadic and inherited forms, and resulting from a complex mix of genetic and vascular risk factors^5,6^. The most common types of sporadic cSVD are the age- and hypertension-related microangiopathy, also called ‘hypertensive arteriopathy’ (HA), and cerebral amyloid angiopathy (CAA)^1,7^. Certainly, CAA and HA have different pathological characteristics. HA refers to arteriolosclerosis, and it predominantly affects small perforating end arteries of the deep white matter and gray nuclei. It is pathologically determined by collagenous thickening of the vessel wall with narrowing of the lumen and progressive loss of smooth muscle. HA is related to ageing, hypertension, and other vascular risk factors such as smoking and type II diabetes^1,6,7^. Progressive deposition of amyloid-β (Aβ) in the walls of cortical and leptomeningeal small arteries is the pathological hallmark of CAA: the vessels become dilated and disrupted, with focal wall fragmentation and blood extravasation, with or without microaneurysmal dilatation, and sometimes show luminal occlusion^8,9^. CAA-related vasculopathy is considered a predisposing factor for vascular occlusion or rupture, and thus ischemic or hemorrhagic parenchymal brain injury^7,10^.

Neuroimaging is nowadays fundamental in the diagnosis of cSVD subtypes which is mainly based on lesion types, location and characteristics. Evidence suggests that intracerebral haemorrhage (ICH) and CMBs in lobar regions are consistently associated with CAA, whereas lacunar stroke, CMBs and ICH in deep regions are associated with HA^7^. Likewise, cSS is frequent in symptomatic CAA, while it is rare in HA^8^.

Also, from a clinical point of view, the two main subtypes of cSVD are characterized by different phenotypes. Generally speaking, both types are associated with stroke, cognitive decline and disability, but other clinical features may help to distinguish the two forms. Typically, CAA is clinically characterized also by transient focal neurological episodes (TFNE)^11^. HA typical clinical manifestations are motor, gait, and mood disturbances.

Cognitive decline caused by cSVD is known as one of the main causes of vascular cognitive impairment, that is believed to present in a stepwise and gradual pattern, mainly characterized by attention and executive functions deficits^1,11–14^.

Cognitive profile is the object of the present manuscript. The following paragraph is a summary of the literature review on the topic.

### Literature review

In MRI studies, functional networks associated with attention and executive functions have been found to be predominantly affected in cSVD patients^6^. This dysexecutive profile is mainly characteristic of cognitive impairment due to hypertension-related microangiopathy, while CAA cognitive profile seemed to be more heterogeneous and it has not yet been well characterized^1,9,12^. Previous studies have shown that moderate to severe β-amyloid depositions in cerebral arterioles seem to be associated with decline in global cognition^15^ and multiple cognitive abilities (perceptual speed, episodic and semantic memory, attention and executive functions)^9,16^. Processing speed seems to be the most severely impaired domain in these patients, followed by executive functions, which is mostly in line with previous findings in vascular cognitive impairment^9,17–19^. Some studies have debated that memory is not severely affected in patients with CAA^9,18,19^. However, even in the absence of large and destructive hematomas, accumulating evidence suggests that CAA is associated with the typical features of neurodegeneration, i.e. brain atrophy and deficits in episodic memory, a hallmark of Alzheimer Disease (AD)^17,20^. Although some degree of overlapping with AD pathology, the cortical thinning and the profile of cognitive impairment that characterized CAA patients suggest the existence of some CAA-specific features of neurodegeneration^20^. The severe cognitive impairment often found in CAA patients may be an indicator of underlying presence of mixed pathology^19^. A recent study found that the most impaired domain was general intellectual functioning, thus hypothesizing that CAA pathology might initially have a generalized, rather than focal, impact on cognitive functions^19^. This hypothesis is in line with the fact that cSVD such as CAA may primarily compromise general intellectual abilities, and this may be in keeping with the pathophysiological process of cSVD which is thought to be widespread, causing disruption to structural and functional white matter networks^19^.

Thus, CAA is increasingly recognized as a cause of cognitive impairment, but its cognitive profile needs to be further characterized. There is recent debate in the literature about the characteristics of cognitive impairment in patients with CAA, which appear to be different from those observed in patients with HA. Several studies conclude that new evidence is needed to better describe the cognitive profile of CAA patients and that neuropsychological assessment should be a standard part of clinical care of these patients. Therefore, our study aims to compare the cognitive profile of HA and CAA patients with the purpose of testing their difference or similarity in terms of cognitive, clinical and functional features.

## Methods

### VAS-Cog clinical approach

The study population was retrospectively selected among patients attending the Florence VAS-Cog Clinic. The Florence VAS-Cog clinic is a reference outpatient service devoted to the management of cognitive, psychiatric, and behavioral disturbances of patients with cerebrovascular diseases. Detailed personal and family history, general and neurologic examinations, and functional, neuropsychological, and neuroimaging exams are routinely evaluated^21,22^ and these patients are followed over time.

The study was conducted in accordance with the Helsinki Declaration, the protocol was approved by the Ethics Committee of the Careggi University Hospital (Florence, Italy), and all participants gave written informed consent.

Among the patients seen in the outpatient clinic, 2 cohorts of patients were retrospectively identified. We initially identified patients with diagnosis of probable or possible CAA defined according to modified Boston neuroradiology criteria (Probable CAA: multiple hemorrhages (ICH, CMBs) restricted to lobar, cortical, or cortical–subcortical regions (cerebellar hemorrhage allowed), or single lobar, cortical, or cortical–subcortical hemorrhage and focal or disseminated cSS; age ≥ 55 years; absence of other cause of hemorrhage. Possible CAA: single lobar, cortical, or cortical–subcortical haemorrhage or focal or disseminated superficial siderosis; age ≥ 55 years; absence of other cause of haemorrhage), and with the ability to perform an extensive second-level cognitive assessment (CAA cohort)^23^.

Secondly, among VAS-Cog patients, we retrospectively identified a group of patients with hypertension-related microangiopathy (HA cohort). More specifically, the inclusion criteria for the HA cohort were the following: evidence of moderate-severe degrees of WMH on MRI, according to the modified version of the Fazekas scale, mild cognitive impairment (MCI) defined according to Winblad et al. criteria and operationalized according to Salvadori et al., and history of ischemic stroke^24,25^. These criteria were selected to avoid the inclusion of patients with covert cSVD. The HA cohort was selected in order to be numerically homogeneous with respect to the CAA cohort.

Each patient underwent an extensive clinical, cognitive, and functional assessment, and brain MRI exam as part of the Florence VAS-Cog outpatient clinic routinely assessment. Neuroimaging characteristics were evaluated on available MRI exams.

### Clinical protocol

All patients were evaluated by a standardized protocol in order to systematically collect socio-demographic (age, years of education, and sex) and clinical data such as: previous cerebrovascular events (ischemic and hemorrhagic stroke), transient focal neurological episodes (i.e., transitory disturbances in motor, somatosensory, visual, or language functions), psychiatric disorders, vascular risk factors (hypertension, dyslipidemia, diabetes, smoking habits, alcohol consumption, ischemic heart disease)^21,22^.

### Cognitive and functional protocol

Cognitive performances of each patient was evaluated by means of a neuropsychological battery that included a global cognitive functioning test (Montreal Cognitive Assessment—MoCA) and five second level tests which cover the following cognitive domains: orientation, verbal memory (Immediate and delayed Rey Auditory Verbal Learning Test—RAVLT), attention, executive functions, information processing speed (Short Stroop Test, Symbol Digit Modalities Test—SDMT), and language (Phonemic and Semantic Verbal Fluency Tests)^21,22,25^.

To evaluate cognitive performance, raw scores have been demographically adjusted and converted into an ordinal 5-point scale (equivalent score, ES) using national normative data. Equivalent score is a non-parametric norming method based on percentiles distributions, and scores range varies from 0 to 4: ES = 0 corresponds to an impaired performance (i.e. adjusted score below the outer confidence limit for the 5th centile of the normal population); ES = 1 corresponds to a borderline performance (i.e. adjusted score between the outer and inner confidence limits for the 5th centile of the normal population); and ES = 2–4 represents a normal performance (i.e. adjusted score above the inner confidence limit for the 5th centile of the normal population). ES methodology was available for all the tests included in the battery except for the Symbol Digit Modalities Test, then its performance was classified as “normal” when the adjusted score was above the 5th centile or “abnormal” when the adjusted score was below the 5th centile of the normal population.

We classified cognitive impairment as at least one borderline score (ES ≤ 1) among the seven scores deriving from the five second level cognitive tests included in the neuropsychological battery. As detailed below, cognitive impairment was also classified according to the MCI subtypes^24^:

- Amnestic (≥ 1 impaired memory test, ES = 0) *vs.* Non-amnestic MCI (none impaired memory test),
- Single (only one impaired domain, ES ≤ 1) *vs.* Multi-Domain MCI (≥ 2 borderline domains, ES ≤ 1).

Functional status was measured by means of activities of daily living scale (ADL, number of preserved items, score range 0–6, higher scores represent less disability) and instrumental activities of daily living scale (IADL sum of scores, score range 0–22, higher scores represent less disability)^26,27^. Depressive symptoms were investigated by Geriatric Depression Scale, 15-item version (GDS, score range 0–15, higher scores represent more severity of depressive symptomatology)^28^.

### Neuroimaging protocol

To make CAA and HA diagnosis, the following neuroimaging markers were visually assessed by a trained and experienced rater using validated scales based on the STRIVE (Standards for Reporting Vascular Changes on Neuroimaging) neuroimaging guidelines^3^: (1) white matter hyperintensities (WMH) were rated on axial FLAIR sequences by means of the Fazekas scale (mild: single lesions < 10 mm; areas of ‘grouped’ lesions < 20 mm in any diameter; moderate: single hyperintense lesions between 10 to 20 mm, areas of ‘grouped’ lesions ≥ 20 mm in any diameter or no more than ‘connecting bridges’ between individual lesions; severe: single lesions or confluent areas of hyperintensity ≥ 20 mm in any diameter)^29^; (2) cerebral microbleeds (CMBs) were assessed according to the Microbleeds Anatomical Rating Scale (MARS) on axial gradient-echo T2* sequences^30^; (3) the same sequences were used to identify all lobar and non-lobar hemorrages as well as cortical superficial siderosis defined as focal (restricted to ≤ 3 sulci) or disseminated (≥ 4 sulci)^31^.

### Statistical analysis

Descriptive statistics (frequencies and percentages, means and standard deviations) were used to describe the total cohort in terms of demographics, vascular risk factors, comorbidities, history of stroke, and cognitive and functional characteristics.

Univariate analyses (independent samples *t* test or chi-square test) were used to compare the CAA and HA cohorts in terms of demographics, vascular risk factors, comorbidities, history of stroke, and cognitive and functional characteristics. For the comparison analyses on cognitive tests, a Bonferroni correction for multiple comparisons was also applied, and the resulting p-value was set at 0.007 (corresponding to the standard p value, 0.05, divided for the 7 tests included in the analysis).

Univariate correlation analyses (Pearson’s r and point-biserial rpb) were conducted separately for CAA or HA patients to evaluate the association between cognitive tests’ performances and demographic, clinic, cognitive and functional characteristics. Multivariate linear regression models were used to evaluate the independent association between cognitive performances and those variables which were significantly associated in univariate correlation analyses. Considering that CAA inclusion criteria did not require the presence of MCI, to reduce the impact of a potential selection bias, univariate between groups and correlation analyses were repeated removing subjects without cognitive impairment from the CAA cohort.

All analyses were done using the SPSS software version 27.

## Results

**Figure 1:**
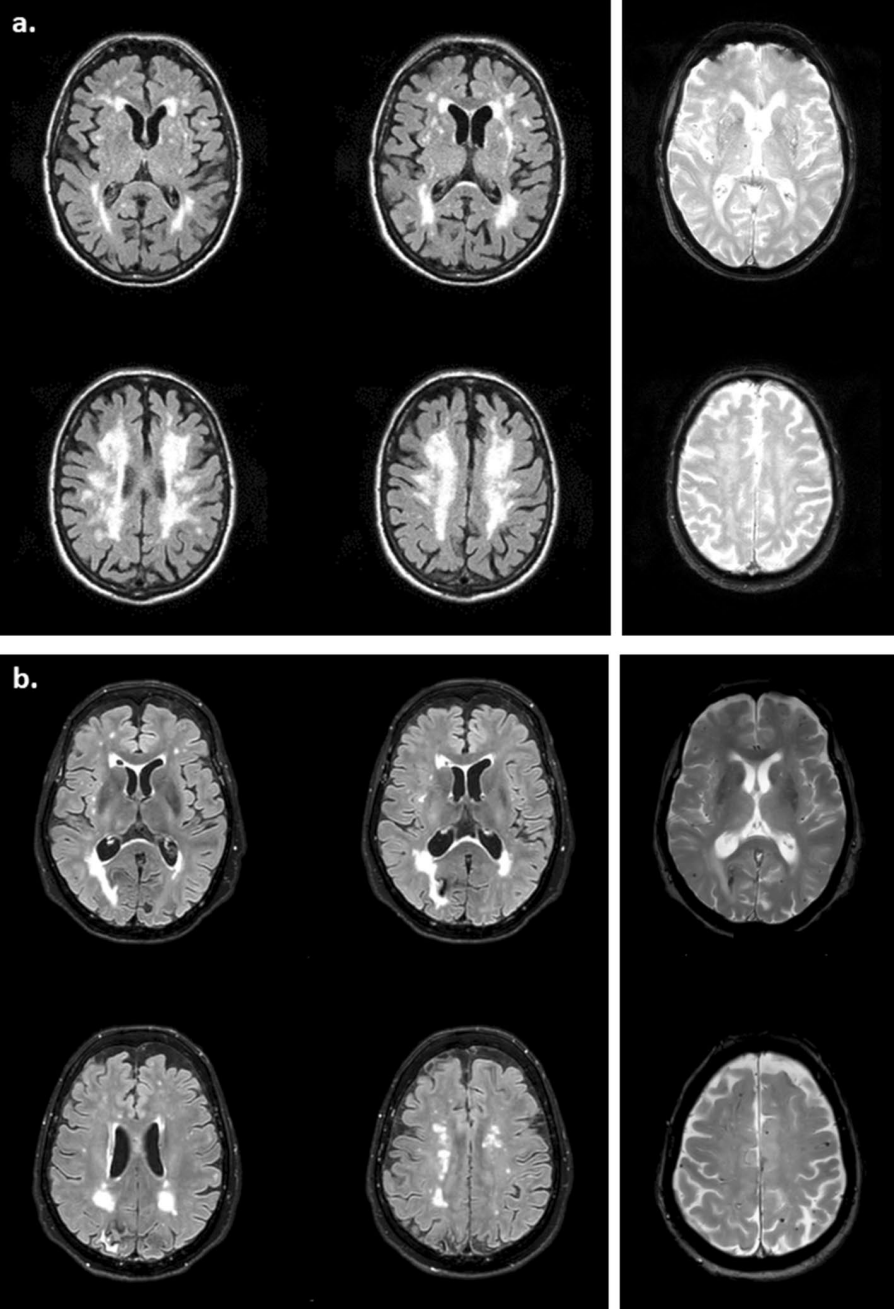
Examples of representative brain of HA and CAA patients. (**a**) Example of a HA patient: Man, 88 years old. History of lacunar stroke three years before; MCI diagnosis according to neuropsychological evaluation. His Brain MRI shows severe subcortical and periventricular WMH (Fazekas 3) in FLAIR sequences. T2* shows one right deep thalamic CMB, with no evidence of lobar CMBs. (**b**) Example of a CAA patient: Woman, 78 years old. History of lobar ICH two years before (right occipital); CAA diagnosis according to modified Boston criteria. Her Brain MRI shows multiple subcortical WMH spots (Fazekas 2) in FLAIR sequences. T2* shows several lobar CMBs, with no evidence of deep CMBs.

Among 484 patients seen in the outpatient clinic from January 2018 to October 2021, we identified 48 patients who received probable or possible CAA diagnosis, of whom 32 patients completed the neuropsychological evaluation (CAA cohort: mean age 76 ± 5.8 years, 47% males). In the CAA cohort, 25 (78%) patients had history of stroke (n = 24 haemorrhagic, n = 1 ischemic), and TFNE were present in 11 (34%) patients. Thirty-nine patients with MCI, moderate-severe white matter hyperintensities and previous stroke (n = 39 ischemic, none haemorrhagic) were included in the HA cohort (mean age 74.1 ± 7.2 years, 74% males). Regarding neuroimaging characteristics, CMBs were present in 23 (79%) CAA patients and in 10 (27%) HA patients. Specifically, 20 CAA patients had lobar CMBs. Representative brain of HA and CAA patients were shown in Fig. 1: the brain MRI of a HA patient, with history of lacunar stroke three years before and MCI diagnosis, shows severe subcortical and periventricular WMH and one right deep thalamic CMB, with no evidence of lobar CMBs; instead the brain MRI of a CAA patient, with history of lobar ICH two years before (right occipital) and CAA diagnosis according to modified Boston criteria, shows multiple subcortical WMH spots and several lobar CMBs, with no evidence of deep CMBs (Fig. 1).

Demographics and clinical characteristics of the total cohort (n = 71 patients, mean age 74.9 ± 6.6 years, 62% males) and comparisons between the two groups are shown in Table 1. There were no significant differences between the CAA and HA cohorts for demographics, except for sex (males 47% *vs.* 74%, respectively, p = 0.018). The presence of vascular risk factors was greater in the HA cohort compared to CAA cohort, in particular dyslipidemia (69% *vs*. 44%, respectively, p = 0.031), alcohol consumption (44% *vs*. 12.5%, respectively, p = 0.004) and smoking habits (49% *vs*. 23%, respectively, p = 0.025).

**Table 1:** Demographics characteristics in the total cohort and comparisons between CAA and HA patients.

	Total cohort	CAA	HA	*P*
	N = 71	N = 32	N = 39	
Age, years	74.9 ± 6.6	76 ± 5.8	74.1 ± 7.2	0.241*
Years of education	7.8 ± 3.6	8.3 ± 3.7	7.4 ± 3.6	0.285*
Sex (males)	44 (62%)	**15 (47%)**	**29 (74%)**	**0.018**^**#**^
Psychiatric disorders	30 (42%)	13 (41%)	17 (44%)	0.801^#^
Hypertension	60 (86%)	24 (77%)	36 (92%)	0.077^#^
Diabetes	5 (7%)	1 (3%)	4 (10%)	0.243^#^
Dyslipidemia	41 (58%)	**14 (44%)**	**27 (69%)**	**0.031**^**#**^
Alcohol consumption	21 (30%)	**4 (12.5%)**	**17 (44%)**	**0.004**^**#**^
Smoking habits	26 (37%)	**7 (23%)**	**19 (49%)**	**0.025**^**#**^
Ischemic heart disease	7 (10%)	3 (9%)	4 (10%)	0.901^#^

Significant values are in bold.

*Independent sample t tests.

^#^Chi square test.

**Figure 2:**
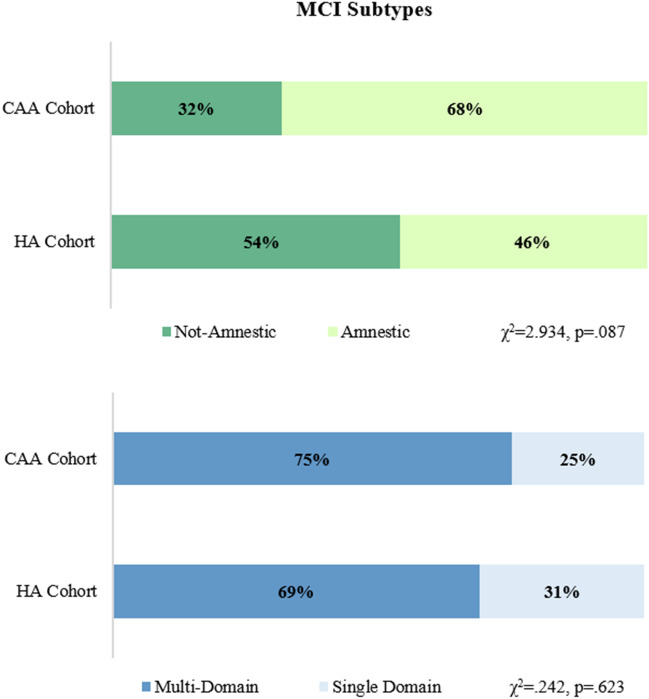
MCI subtypes distributions: comparisons between CAA and HA patients (χ^2^ tests).

**Figure 3:**
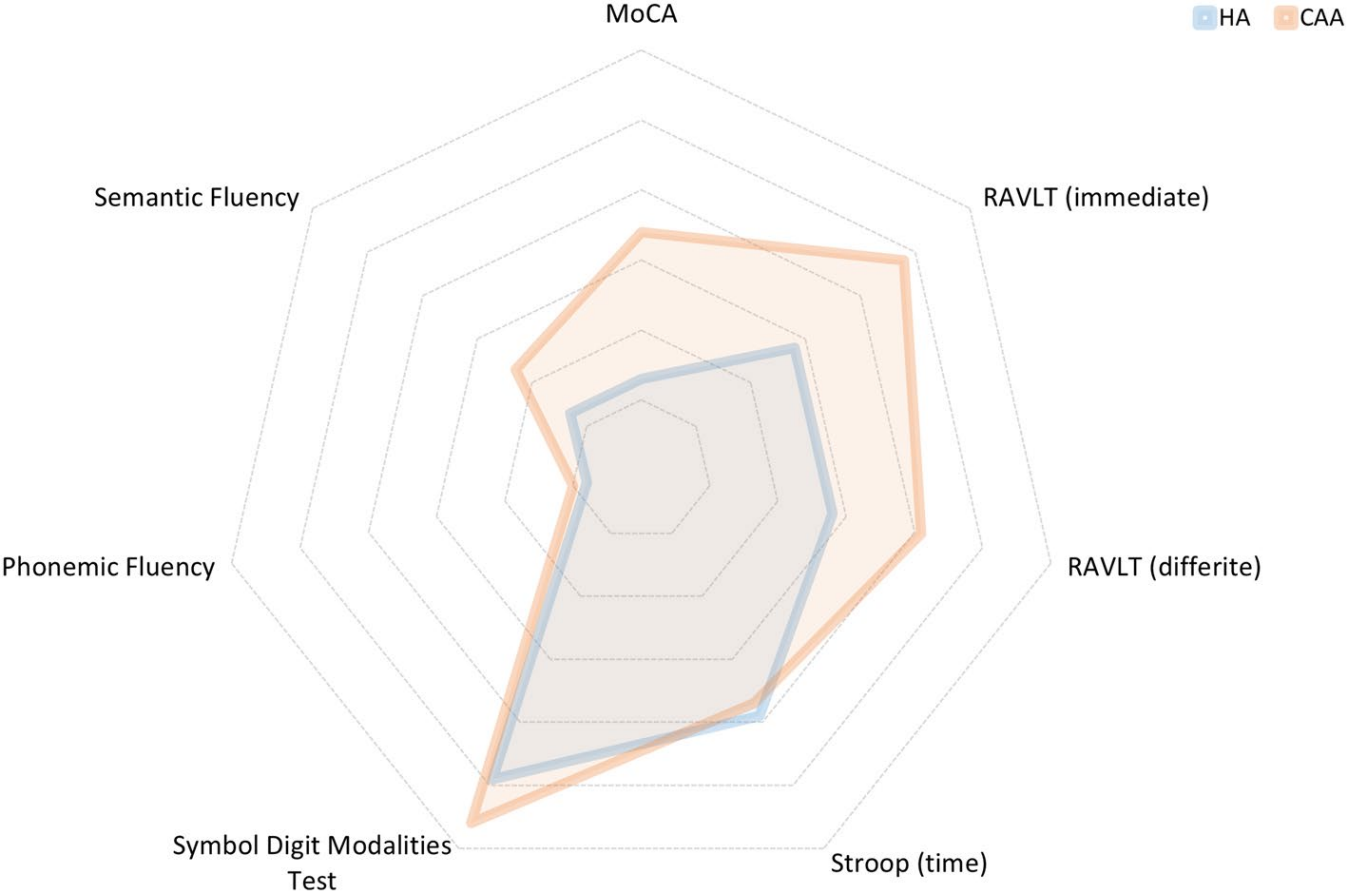
Graphical representation of the cognitive domains impaired in patients with CAA and HA. The graph shows the percentages of patients who had obtained PE = 0 (impaired performance) on each neuropsychological test in the CAA and HA cohorts.

As shown in Table 2, from a cognitive point of view, compared to HA patients, CAA ones presented a worse performance at MoCA (mean score 21.5 ± 4.1 *vs.* 17.6 ± 5.4, respectively, p < 0.001) and semantic fluency (mean score 35.2 ± 6 *vs.* 30.7 ± 10.7, respectively, p = 0.043). The diagnosis of cognitive impairment was present in 28 CAA patients (87.5%) and in all HA ones by definition. As shown in Fig. 2, the amnestic MCI subtype was more frequent in CAA patients compared to HA ones (68% *vs.* 46%, respectively, p = 0.087). Subgroups analyses confirmed previous results and further highlighted that, compared to HA cohort, CAA patients with cognitive impairment had a significantly worse performance at Symbol Digit Modalities Test (mean score 36.3 ± 9 *vs.* 31.7 ± 6.4, respectively, p = 0.029) and a trend was found also for Phonemic Fluency (mean score 29.2 ± 10.4 *vs.* 24.9 ± 7.6, respectively, p = 0.061) (Table 3). After Bonferroni correction for multiple comparisons, the statistically significant reduction in global cognitive efficacy (MoCA) in CAA patients compared to HA ones was confirmed. As shown in Fig. 3, CAA patients had more complex and generalized cognitive impairment than HA patients (Fig. 3).

**Table 2:** Cognitive and functional characteristics in the total cohort and comparisons between CAA and HA patients.

	Total cohort	CAA	HA	*p*
	N = 71	N = 32	N = 39	
Montreal cognitive assessment (score range 0–30)	19.8 ± 5.1	**17.6 ± 5.4**	**21.5 ± 4.1**	0.001*
Rey auditory verbal learning test (immediate)	33.5 ± 8.6	32.6 ± 9.1	34.1 ± 8.2	0.495*
Rey auditory verbal learning test (differite)	6.2 ± 2.6	6.2 ± 2.7	6.2 ± 2.6	0.944*
Stroop test (time)	38.6 ± 30.9	36.4 ± 32.9	40.3 ± 29.7	0.619*
Symbol digit modalities test	35.4 ± 8.6	33.8 ± 7.9	36.3 ± 9	0.267*
Phonemic verbal fluency test	28.2 ± 10	26.8 ± 9.4	29.2 ± 10.4	0.327*
Semantic verbal fluency test	33.3 ± 8.6	**30.7 ± 10.7**	**35.2 ± 6**	0.043*
Geriatric depression scale (range 0–15)	4.1 ± 3	5 ± 3.3	3.7 ± 2.9	0.115*
ADL (preserved items, range 0–6)	5.5 ± 0.9	5.5 ± 0.9	5.6 ± 0.9	0.526*
IADL (total score, range 0–22)	16.4 ± 5	15.5 ± 5.9	17.2 ± 4.2	0.152*

Significant values are in bold.

*Independent sample t tests.

^#^Chi square test.

**Table 3:** Comparisons of cognitive and functional characteristics between CAA and HA patients, excluding patients without cognitive impairment (subgroups analyses).

	CAA	HA	*p*
	N = 28	N = 39	
Montreal cognitive assessment (score range 0–30)	**16.5 ± 4.7**	**21.5 ± 4.1**	0.001*
Rey auditory verbal learning test (immediate)	30.87 ± 8.0	34.1 ± 8.2	0.126*
Rey auditory verbal learning test (differite)	5.5 ± 2.1	6.2 ± 2.6	0.223*
Stroop test (time)	39.5 ± 34.7	40.3 ± 29.7	0.928*
Symbol digit modalities test	**31.7 ± 6.4**	**36.3 ± 9**	0.029*
Phonemic verbal fluency test	24.9 ± 7.6	29.2 ± 10.4	0.061*
Semantic verbal fluency test	**29.0 ± 10.4**	**35.2 ± 6**	0.003*
Geriatric depression scale (range 0–15)	5 ± 3.2	3.7 ± 2.9	0.144*
ADL (preserved items, range 0–6)	5.5 ± 1.0	5.6 ± 0.9	0.647*
IADL (total score, range 0–22)	14.8 ± 6.1	17.2 ± 4.2	0.078*

Significant values are in bold.

*Independent sample t tests.

^#^Chi square test.

Univariate correlation analyses (Table 4), conducted separately for CAA or HA patients, showed that the multi-domain MCI subtype was associated with a worse performance at MoCA (r_pb_ = − 0.431, p = 0.036), immediate (r_pb_ = − 0.544, p = 0.007) and delayed (r_pb_ = − 0.549, p = 0.007) RAVLT, and semantic fluency (r_pb_ = − 0.430, p = 0.036) in CAA patients. In HA cohort the multi-domain MCI subtype was associated with a worse performance at SDMT (r_pb_ = − 0.364, p = 0.023) and phonemic fluency (r_pb_ = − 0.351, p = 0.029). In CAA patients, functional status measured by instrumental activities of daily living (IADL) was associated with worse global cognitive efficiency (MoCA: r_pb_ = 0.607, p = 0.001), verbal memory (immediate RAVLT: r_pb_ = 0.567, p = 0.001, and delayed RAVLT: r_pb_ = 0.601, p = 0.001), and semantic fluency (r_pb_ = 0.614, p = 0.001), while basic functional status (Activities of Daily Living) was associated with worse global cognitive efficiency (MoCA: r_pb_ = 0.477, p = 0.007). Depressive symptoms (GDS score) were found to be associated with a worse performance at MoCA (r_pb_ = − 0.353, p = 0.028) in the HA cohort, while no significant associations were found with functional status. Subgroups analyses including only CAA patients with cognitive impairment showed that also the basic functional status (ADL) was associated with verbal memory (immediate RAVLT: r_pb_ = 0.431, p = 0.031), and semantic fluency (r_pb_ = 0.470, p = 0.015) (Table 5).

**Table 4:** Association between cognitive tests’ performances and demographic, clinic, cognitive and functional characteristics separately for CAA or HA patients.

			Age	Education	Sex	Single/Multi domain	ADL	IADL	GDS
CAA Cohort	MoCA	Univariate	− 0.167	0.177	0.027	− 0.431*	0.477*	0.607*	− 0.052
		Multivariate				ns	ns	0.539*	ns
	RAVLT (immediate)	Univariate	− 0.147	0.173	0.253	− 0.544*	0.307	0.567*	− 0.214
		Multivariate				− 0.574*	ns	ns	ns
	RAVLT (delayed)	Univariate	− 0.318	0.079	0.247	− 0.549*	0.212	0.601*	− 0.134
		Multivariate				ns	ns	0.645*	ns
	Stroop (time)	Univariate	0.219	− 0.127	− 0.066	0.311	0.057	− 0.162	− 0.013
		Multivariate				0.491	ns	ns	ns
	SDMT	Univariate	− 0.250	− 0.125	− 0.103	− 0.294	0.143	0.296	− 0.105
		Multivariate				ns	0.542*	0.455	− 0.532
	Phonemic Fluency	Univariate	− 0.030	− 0.199	0.239	− 0.263	− 0.098	0.214	0.193
		Multivariate				ns	ns	ns	ns
	Semantic Fluency	Univariate	− 0.231	0.166	0.154	− 0.430*	0.336	0.614*	0.235
		Multivariate				ns	ns	0.541*	ns
HA Cohort	MoCA	Univariate	0.028	0.069	0.064	− 0.067	0.130	0.300	− 0.353*
		Multivariate				ns	ns	ns	− 0.353*
	RAVLT (immediate)	Univariate	− 0.182	0.024	0.244	− 0.252	0.096	0.040	0.081
		Multivariate				ns	ns	ns	ns
	RAVLT (delayed)	Univariate	0.076	0.140	− 0.055	− 0.170	− 0.153	− 0.069	0.163
		Multivariate				ns	ns	ns	ns
	Stroop (time)	Univariate	0.149	− 0.287	− 0.239	0.175	− 0.162	− 0.304	− 0.094
		Multivariate				ns	ns	− 0.304	ns
	SDMT	Univariate	0.039	0.048	0.038	− 0.364*	0.115	0.188	0.066
		Multivariate				− 0.364*	ns	ns	ns
	Phonemic fluency	Univariate	− 0.121	− 0.024	0.332	− 0.351*	− 0.060	0.151	− 0.114
		Multivariate				− 0.351*	ns	ns	ns
	Semantic fluency	Univariate	− 0.318*	− 0.014	− 0.040	− 0.316	0.006	0.014	0.100
		Multivariate				− 0.316	ns	ns	ns

Significant values are in bold.

Univariate models: Pearson’s r and point-biserial rpb correlation analyses.

Multivariate models: linear regressions adjusted for age, education and sex.

*p < 0.050.

**Table 5:** Association between cognitive tests’ performances and demographic, clinic, cognitive and functional characteristics for CAA patients with cognitive impairment (subgroups analyses).

			Age	Education	Sex	Single/Multi domain	ADL	IADL	GDS
CAA Cohort	MoCA	Univariate	− 0.139	0.284	− 0.071	− 0.431*	0.645**	0.595**	− 0.191
		Multivariate				ns	ns	0.539*	ns
	RAVLT (immediate)	Univariate	0.015	0.121	0.241	− 0.544**	0.431*	0.522**	− 0.029
		Multivariate				− 0.574*	ns	ns	ns
	RAVLT (delayed)	Univariate	− 0.216	0.146	0.334	− 0.549**	0.317	0.595**	− 0.083
		Multivariate				ns	ns	0.645*	ns
	Stroop (time)	Univariate	0.221	− 0.159	− 0.049	0.311	0.022	− 0.106	− 0.007
		Multivariate				0.491	ns	ns	ns
	SDMT	Univariate	− 0.198	− 0.013	− 0.122	− 0.294	0.398	0.185	− 0.243
		Multivariate				ns	0.542*	0.455	− 0.532
	Phonemic fluency	Univariate	− 0.023	− 0.228	0.118	− 0.263	0.068	0.123	0.004
		Multivariate				ns	ns	ns	ns
	Semantic fluency	Univariate	− 0.186	0.245	0.114	− 0.430*	0.470*	0.580**	0.250
		Multivariate				ns	ns	0.541*	ns

Significant values are in bold.

Univariate models: Pearson’s r and point-biserial rpb correlation analyses.

Multivariate models: linear regressions adjusted for age, education and sex.

*p < 0.050.

**p < 0.01.

**Figure 4:**
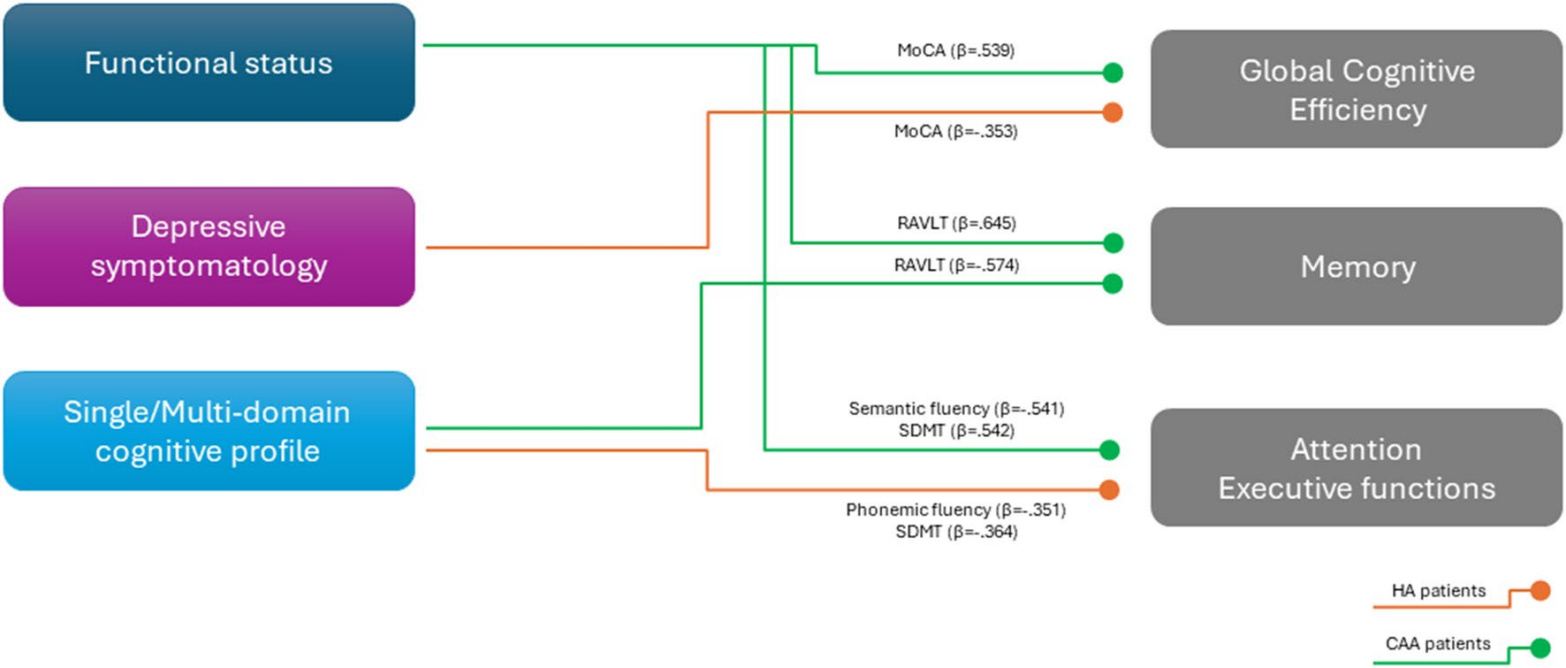
Graphical representation of multivariate logistic regression models computed including single/multi-domain MCI, ADL, IADL and GDS as independent variables and adjusting for age, education and sex. Green arrows refer to the CAA cohort, while orange arrows refer to the HA cohort.

Multivariate logistic regression models were computed including single/multi-domain MCI, ADL, IADL and GDS as independent variables and adjusting for age, education and sex (Table 4). The presence of a multi-domain deficit was confirmed as a factor negatively associated with performance at immediate RAVLT (β = − 0.574, p = 0.032) in CAA cohort and with performance at SDMT (β = − 0.364, p = 0.023) and phonemic fluency (β = − 0.351, p = 0.029) in HA cohort. Functional status remained as the only factor independently associated with MoCA (β = 0.539, p = 0.038), verbal memory (delayed RAVLT: β = 0.645, p = 0.013) and semantic fluency (β = − 0.541, p = 0.037) in CAA patients. In addition, functional status emerged as a factor independently associated with performance at SDMT (β = 0.542, p = 0.023) in CAA cohort. In HA cohort, the depressive symptomatology was confirmed as independently associated with global cognitive efficiency (β = − 0.353, p = 0.028). Figure 4 graphically illustrates the results obtained at the multivariate analyses (Fig. 4).

## Discussion

The main purpose of the present study was the comparison of the cognitive profiles between CAA and HA cSVD patients. We also compared clinical and functional features to investigate differences and similarities between the two majors sporadic cSVD forms.

Overall, in our population 87.5% of CAA patients received a diagnosis of cognitive impairment, thus confirming the high rate of cognitive decline in this patients’ population already reported in previous studies^17^.

Compared to HA cSVD patients, CAA ones presented a reduced global cognitive efficiency, a higher rate of the amnestic MCI subtype, and a multi-domain profile of cognitive deficits that included verbal memory and semantic elaboration, as well as executive functions, attention and information processing speed^9,16,17,32^. In CAA patients the presence of a multi-domain impairment could represent a marker of a higher cognitive burden and involved also those cognitive domains expected to be impacted by neurodegenerative mechanisms. Our results are consistent with what has been found in the literature, both from the perspective of the complexity of the overall cognitive profile of CAA patients^19,33^ and by focusing on individual domain-specific deficit functions^16,17^. Conducting the analysis excluding patients without cognitive impairment, it is interesting to note that information processing speed is more impaired in CAA patients, as found in previous studies^9,17–19^.

On the other side, in HA patients the not-amnestic subtype was confirmed as the main cognitive profile, and the presence of a multi-domain cognitive impairment was associated with decreased attentional and executive performance^12^. Overall, our results seem to support a greater variability of cognitive manifestations in CAA, unlike a more homogeneous characterization of deficits associated with HA microangiopathy, which have a more representative and defined cognitive profile^12,17^. Furthermore, our findings highlighted that CAA patients seem to be more functionally compromised in both basic and instrumental daily activities compared to HA ones^17^.

With respect to clinical features, in line with previous evidence that showed some differences on clinical characteristics of the two diseases due to involvement of different pathological mechanisms, a higher burden of vascular risk factors was found in HA patients compared to CAA ones. In line with this, the frequency of vascular risk factors in the HA cohort is likely to also determine the higher rate of males^34^. Overall, CAA patients seem to be more cognitively and functionally impaired than HA subjects, and our results further highlight the complexity and heterogeneity of the clinical and cognitive profile in CAA patients. Interestingly, our results are in line with the hypothesis that CAA pathology may have a role in mixed dementia, due to the presence of both neurodegenerative and vascular underlying mechanisms as suggested by Ramusino and colleagues^32^.

The present study has several limitations that need to be highlighted. First, limited sample size and retrospective study design are major shortcomings. The small sample size negatively affected statistical power, and thus reduced generalizability of our results. The retrospective study design represents another limitation of this study. Further studies based on prospective design and longitudinal data acquisition are needed to better characterize profiles of cognitive deficits in sporadic cSVD. A second limitation concerns the fact that CAA diagnosis cannot be definitively excluded in HA patients. Updated Boston criteria for the CAA diagnosis were recently published and their application in our cohort would allow to take into account also the spatial patterns of WMHs (e.g. multiple spots vs. peri–basal ganglia) which could further help to differentiate between the two forms^8,35^. In fact, both CAA and HA are associated with WMH, but these two diseases present different patterns of WMH: multiple punctate subcortical hyperintensities are more commonly found in CAA, while peri-basal ganglia confluent WMH are more common in HA^8^. Moreover, the deposition of cSS was assessed only in the CAA cohort, as per the diagnostic criteria, but not in the HA cohort. Among new and advanced neuroimaging techniques, in future studies it would be interesting to use the technique of Quantitative Susceptibility mapping (QSM). This method has been widely used for the quantification of cSS and CMBs in cSVD and it allow the detection of a higher number of CMBs and the differentiation of CMBs from calcifications^36,37^. A third limitation pertains the possibility that our inclusion criteria could results in potential selection biases. On one side, differently from the HA cohort, the CAA one also included patients without cognitive impairment, as its presence was not required in CAA diagnostic criteria. On the other side, the HA cohort enrollment was not consecutive. Furthermore, despite other qualitative measures would be more precise, HA enrollment was based on the Fazekas scale. The choice of this scale was based on the fact that it is routinely applied and available, reflecting the real-world clinical setting of our VAS-Cog clinic. From the cognitive point of view, we decided to limit our study to patients with MCI that represents only the initial stages along the vascular cognitive impairment (VCI) continuum^12^. This choice was aimed at potentially identifying distinctive patterns of cognitive deficits that could be more evident in prodromal phases. Finally, in the context of cerebrovascular diseases, the use of the original MCI diagnostic algorithm may be partially misleading. Winblad criteria for MCI are hierarchically oriented toward the identification of amnestic deficits prior to decline in other cognitive functions, thus resulting in an underestimation of the impact of the attentional and executive deficits^24^. For this reason, in addition to using MCI subtypes according to Winblad’s criteria, it would be interesting to explore the presence of prevalent, and/or potentially distinguishable, cognitive profiles defined according to either the most impaired cognitive functions, or an empirical data-driven approach.

## Conclusion

In conclusion, the present study seems to confirm the existence of two potentially different patterns of cognitive deficits in CAA or HA patients. Specifically, while the cognitive profile of HA patients was confirmed as mainly attentional/executive, CAA patients seem to have a more complex and heterogeneous cognitive profile, characterized by reduced global cognitive efficiency and deficit in semantic memory.

## Data Availability

The data that support the findings of this study are available on request from the corresponding author. The data are not publicly available due to privacy or ethical restrictions.
