# Author Correction: Cognitive profile in cerebral small vessel disease: comparison between cerebral amyloid angiopathy and hypertension-related microangiopathy

**DOI:** 10.1038/s41598-024-59216-y

**Published:** 2024-04-16

**Authors:** Eleonora Barucci, Emilia Salvadori, Simona Magi, Martina Squitieri, Giulio Maria Fiore, Lorenzo Ramacciotti, Benedetta Formelli, Francesca Pescini, Anna Poggesi

**Affiliations:** 1 NEUROFARBA Department, Neuroscience Section, University of Florence, Careggi University Hospital, Largo Brambilla 3, 50134 Florence, Italy; 2 Department of Biomedical and Clinical Sciences, University of Milan, Milan, Italy; 3 Stroke Unit, Careggi University Hospital, Florence, Italy

Correction to: *Scientific Reports* 10.1038/s41598-024-55719-w, published online 11 March 2024

The original version of this Article contained errors in the Affiliations.

Francesca Pescini and Anna Poggesi were incorrectly affiliated with ‘Department of Biomedical and Clinical Sciences, University of Milan, Milan, Italy’ and ‘Don Carlo Gnocchi Foundation, Florence, Italy’.

The correct Affiliations for Francesca Pescini and Anna Poggesi are listed below.

NEUROFARBA Department, Neuroscience Section, University of Florence, Careggi University Hospital, Largo Brambilla 3, 50134 Florence, Italy.

Stroke Unit, Careggi University Hospital, Florence, Italy.

In addition, the Affiliation ‘Don Carlo Gnocchi Foundation, Florence, Italy’ was removed.

The original Article has been corrected.

